# Patient and provider perspectives on using telemedicine for chronic disease management among Native Hawaiian and Alaska Native people

**DOI:** 10.3402/ijch.v72i0.21401

**Published:** 2013-08-05

**Authors:** Vanessa Hiratsuka, Rebecca Delafield, Helene Starks, Adrian Jacques Ambrose, Marjorie Mala Mau

**Affiliations:** 1 Research Department, Southcentral Foundation, Anchorage, AK, USA; 2 Department of Native Hawaiian Health, John A. Burns School of Medicine, University of Hawai'i, Honolulu, HI, USA; 3 Department of Bioethics and Humanities, University of Washington, Seattle, WA, USA

**Keywords:** Alaska, Indians, North America, focus groups, technology use, chronic disease treatment, patient perspective, provider perspective

## Abstract

**Background:**

Among indigenous populations in remote locations who are at increased risk for chronic diseases such as diabetes, telemedicine has the potential to improve access to health care services and thus may reduce adverse health outcomes. Yet few studies are available on how best to use telemedicine technology in reducing ethnic and racial health care disparities.

**Objective:**

We examined perspectives of patients and providers in 2 indigenous populations in Alaska and Hawai'i about the use of telemedicine in primary care chronic disease management.

**Design:**

Six focus groups with patients and providers at 2 sites (3 in Alaska and 3 in Hawai'i).

**Results:**

Three broad themes were common to both sites: (a) benefits and barriers of using telemedicine; (b) building patient–provider relationships; and (c) elements of an acceptable telemedicine primary care encounter. Two key elements were endorsed by both patients and providers as important for an effective telemedicine encounter: (a) the initial patient–provider interaction should be face-to-face; and (b) patients must see the same provider on follow-up visits.

**Conclusion:**

The use of telemedicine in chronic disease management has potential to improve patient care in remote indigenous populations and may supplement patient–provider relationships.

Telemedicine programs are promoted as a solution to improve access to health care for rural, remote and medically underserved communities (1, 2). The high turnover of providers in rural areas and the lack of specialty care services throughout remote rural health care delivery areas have led to a growing interest in telemedicine-supplemented health systems. For rural Native populations in Hawai'i and Alaska, the issue of physician shortages and geographic separation is particularly problematic (3). In Alaska, 23 of 27 of its boroughs and census areas are federally designated as medically underserved areas or populations (4). In Hawai'i, the problem of physician shortages is worsening. A 2010 assessment of the state's shortage showed that Hawai'i has 20% fewer doctors compared to the national physician population ratios, with much of the shortage in primary care (5, 6).

Limited access to health care is of particular concern because chronic diseases, such as diabetes, heart disease and hypertension disproportionately affect racial/ethnic minority populations that often reside in remote communities (7). Thus, the potential of telemedicine to address these health care disparities is being evaluated as a means to improve access and quality of care for communities in Alaska and Hawai'i (3).

However, for telemedicine to be effective, it must increase access to care while providing health services which simulate key activities for adequate clinical assessment such as a history and physical examination. Patient–provider communication and relationships are important factors influencing health care outcomes (8) and are essential when addressing health care for Native populations. In studies with Native Hawaiian (NH) patients, issues of trust and interpersonal and communication skills are identified as key aspects of culturally competent health care (9–11). A limitation in the existing telemedicine literature is the lack of studies examining cultural competency within encounters facilitated by telemedicine technologies.

In this article, we describe the perspectives about the use of telemedicine in chronic disease management from patients and providers in Alaska Native (AN) and NH communities. This study was conducted in part to better understand the current use of telemedicine at both locations in preparation for developing a clinical intervention to improve diabetes management, as an example of chronic disease management, in rural and medically underserved Native communities.

## Materials and methods

Six focus groups were conducted in Alaska (n=3) and Hawai'i (n=3). Southcentral Foundation (SCF) and Na Pu'uwai assisted with focus group protocols. SCF is an AN-owned and operated health corporation located in Anchorage that provides care through the Anchorage Native Primary Care Center, the Benteh Nuutah Clinic serving the Matanuska-Susitna Valley, as well as 51 sub-regional and village clinics serving more than 60 villages. Na Pu'uwai is the NH health care system on the islands of Molokai and Lanai. Despite their geographic distance, both Native-serving organizations have parallel missions of providing health care to their respective Native communities and/or villages. The health care providers at both sites have a history of using telemedicine as an adjunct to face-to-face clinical encounters.

The types of telemedicine technologies and programs varied considerably within each site's health care system. The telemedicine method most commonly used at the Hawai'i site is “real-time” video teleconferencing, which transmits and portrays data without time latency. In contrast, some providers at the Alaska site had experience using home monitoring units as well as “store and forward” telemedicine devices (12). “Store and forward” involves the recording of clinical data and “storing” the data on a server or device, which later transmits (“forwards”) the information to a tertiary care centre for review by a physician or nurse practitioner. Physician recommendations are then relayed back to the remote site using the same technology.

### Participant recruitment and data collection

At the Alaska site, 3 focus groups were conducted: (a) a group of non-physician health care providers caring for Alaska Native/American Indian (AN/AI) primary care patients; (b) a group of primary care providers and specialists caring for AN/AI people; and (c) a group of AN/AI adults who utilized the Anchorage Native Primary Care Center with self-reported type 2 diabetes. At the Hawai'i site, 3 focus groups were conducted: A group of physician and non-physician primary care providers practicing on Molokai; and 2 groups with patients who reside on the island of Molokai with self-reported type 2 diabetes and/or caregivers of individuals with diabetes. In Alaska, health care providers and patients were recruited directly from the primary care settings using e-mails, phone calls, and posting of flyers at clinic lobbies, work areas, and at AN health and wellness gatherings over a 3-week period of time. At the Hawai'i site, all physicians and non-physician providers on the island of Molokai were invited by personal letters. Six of the seven physicians practicing on Molokai participated in the focus group session. All patient/caregiver participants were recruited through Na Pu'uwai's diabetes education programs over a 4-week period. All participants gave written informed consent prior to attending the focus group sessions.

An experienced researcher at each site moderated the focus groups using the same interview guide. All focus groups were recorded by a scribe and audio recorded for subsequent transcription. Focus group questions elicited participants’ views on using telemedicine technology for health services and comments about the effects of the technology on perceived cultural competency in health care delivery. Participants were asked to give their opinions about the benefits and drawbacks of telemedicine. No single definition of telemedicine was provided in any of the groups. The facilitators or participants themselves offered examples, such as television, telephone, video camera, and computers to: (a) provide a clearer understanding of technologies included in local definitions of telemedicine and, (b) describe participants’ experiences with these technologies in health care settings, including aspects of cultural competency. All focus groups were held in English. Focus groups at the Alaska site were held at the Anchorage Native Primary Care Center, and focus groups at the Hawai'i site were held at the Na Pu'uwai health centre.

All participants completed a short questionnaire about their demographics and prior experience with telemedicine. The Alaska Area Institutional Review Board and local tribal approving bodies approved the Alaska site study and the Hawai'i site study was approved by the University of Hawai'i Institutional Review Board.

### Analysis

Transcripts were coded independently by study staff. Data were analyzed using a thematic network approach (13) to identify common views across the different participant groups about the benefits and drawbacks of using telemedicine in diabetes management. Data codes and themes at each site were inductively generated. After the initial coding was completed, the coders from each site met to compare results. During this joint review, themes that met all of the following criteria were extracted for analysis: (a) mentioned in a single focus group; (b) endorsement or elaboration by another member of the focus group; and (c) mention of similar perspective/issue in more than one group per site. The coders organized the themes (with exemplar quotations) by site, participants and group type (patient or provider) and then reviewed the combined data to identify commonalities and differences between sites and group type.

## Results

### Participant characteristics

Forty individuals participated in the 6 focus groups. Characteristics of the patient (which included caregivers in the Hawai'i focus groups) and provider groups are summarized in Table I. The majority of the patients were either NH or AN/AI (82%), women (76%), aged 45 or older (77%), and had some college education or had graduated from college (76%). Of the health care providers, the majority were physicians (70%), male (57%) and Caucasian (57%). Within the Alaska provider group, only one identified as AN/AI compared to 80% of the patients. Similarly, only 2 providers at the Hawai'i site were identified as NH as compared to 75% of the Hawai'i patients.

**Table I:** Participant characteristics

Patient/caregiver characteristics	Categories	Alaska (n=5)	Hawai'i (n=12)	Total (n=17)
Sex	Female	4 (80%)	9 (75%)	13 (76%)
	Male	1 (20%)	3 (25%)	4 (24%)
Age category	25–34 years	0 (0%)	1 (8%)	1 (6%)
	35–44 years	0 (0%)	3 (25%)	3 (18%)
	45–54 years	1 (20%)	1 (8%)	2 (12%)
	55–64 years	2 (40%)	5 (42%)	7 (41%)
	65+ years	2 (40%)	2 (17%)	4 (24%)
Primary ethnicity/race	American Indian/Alaska Native	5 (100%)	0 (0%)	5 (29%)
	Native Hawaiian	0 (0%)	9 (75%)	9 (53%)
	Caucasian	0 (0%)	2 (17%)	2 (12%)
	Asian (Filipino)	0 (0%)	1 (8%)	1 (6%)
Education level	No high-school diploma	0 (0%)	1 (8%)	1 (6%)
	High-school diploma or GED	0 (0%)	3 (25%)	3 (18%)
	Some college/college graduate	5 (100%)	8 (67%)	13 (76%)
Provider characteristics	Categories	Alaska (n=15)	Hawai'i (n=8)	Total (n=23)
Sex	Female	7 (47%)	3 (38%)	10 (43%)
	Male	8 (53%)	5 (63%)	13 (57%)
Age	25–34 year category	4 (27%)	1 (13%)	5 (22%)
	35–44 year category	5 (33%)	0 (0%)	5 (22%)
	45–54 year category	4 (20%)	1 (13%)	5 (22%)
	55–64 year category	2 (13%)	4 (50%)	6 (26%)
	65+ years category	0 (0%)	2 (25%)	2 (9%)
Primary ethnicity/race	Alaska Native/American Indian	1 (7%)	0 (0%)	1 (4%)
	Native Hawaiian	0 (0%)	2 (25%)	2 (9%)
	Asian	5 (33%)	1 (13%)	6 (26%)
	Caucasian	8 (53%)	5 (63%)	13 (57%)
	Other	1 (7%)	0 (0%)	1 (4%)
Health profession	MD	10 (67%)	6 (75%)	16 (70%)
	RN or PA	1 (7%)	1 (13%)	2 (9%)
	Other	4 (27%)	1 (13%)	5 (22%)
Practice location	Rural	15* (100%)	7 (88%)	7 (88%)
	Urban	15 (100%)	0 (0%)	0 (0%)
				
	Unknown	–	1 (12%)	1 (12%)
Experience using telemedicine technologies to provide medical care	Yes	6 (40%)	4 (50%)	10 (43%)

* All providers serve both urban and rural locations.

Although there was some variation between sites, 3 broad themes were common across participants at both sites: (a) benefits and barriers of using telemedicine for a primary care encounter; (b) building patient–provider relationships; and (c) elements of an acceptable telemedicine-mediated primary care encounter. We elaborate on each of these themes below and provide exemplar quotes to illustrate participants’ perspectives.

### Benefits and barriers to incorporating technology into a primary care encounter

The primary benefit of using telemedicine identified by both patients and providers was the potential of reducing travel and health care costs. For patients, travelling from their home in a rural location to an urban-based health clinic is a potential barrier, even when the cost of the travel is covered by insurance. They cited concerns regarding the travel time, finding housing and ground transportation, and orienting themselves to unfamiliar environments. Providers also noted rising travel costs and lost clinic time when travelling to remote sites. Other benefits include increased access and decreased wait times for patients versus waiting for physicians and specialists to travel to their rural communities to provide care.

Providers spoke more specifically than the patients about the employed types of telemedicine technologies and their experiences in using them. Differences in the forms of technology and connections between provider's clinical settings and remote clinical sites influenced their perceptions of telemedicine's value. Some providers had experienced good connections and images, and therefore considered telemedicine technologies a useful “tool.” However, others experienced suboptimal image clarity, poor or unreliable internet connections, and delays from technical difficulties. Providers at both sites expressed the need for information technology support and dedicated staff with expertise to ensure efficient use of telemedicine.

It works great when it works, but let me tell you, in my clinics, it's more not working than working. And if it is working, the staff doesn't know how to use it. At one of my other clinics, they added on all these new tools, but by the time they [were] added on, [the staff] didn't know quite how to use [it]. And so it's one of those missing links of getting people together to actually get the equipment running and then making sure we have internet access, which is spotty out in … [Rural Alaska areas] … and having the technicians that are capable to run it on a regular basis.

Additional barriers related to the absence of face-to-face contact during the clinical encounter. Both patients and providers articulated some unease about the lack of physical contact and hands-on interaction when using telemedicine technologies. Patients focused on issues of non-verbal communication and personal “connection” or the need to develop a relationship between the patient and the provider. Providers focused on the potential for “missing something” medical when conducting a thorough physical examination remotely. A primary care physician at the Hawai'i site stated:

Being a little old fashioned, I have problems with doing things like physical diagnosis over a television set … my experience with video teleconference is that there is always something lost when you do this. You lose some of the non-verbal signals … it took me years to realize how important those are.

The degree to which physical distance affected providers’ views on telemedicine seemed to depend on personal experiences, practice area and type of technology used. One provider from Hawai'i, who has more experience in using video teleconferencing to conduct visits with patients remotely, commented:

I think one of our frustrations with the dermatology [consult] was … [w]e didn't feel like we always got good quality pictures and there wasn't that ability to sort of touch somebody… . But I, I do think that in primary care an amazing part of what we do is the talking part of the visit. And I feel that I spend a lot of time doing what I think of as education as much as anything else. And I think that the telemedicine is really quite adaptable to that kind of visit. And I think especially when we are talking about managing chronic diseases, there's a huge amount of just chit chat that is a part of that and there's certainly no reason why that can't be done over telemedicine.

Barriers to the use of telemedicine also included technical difficulties with the technology. Regarding the variety of available technologies to provide health care remotely combined with the needed medical care of these rural and underserved communities, providers had mixed views about the utility of telemedicine as a tool for helping them care for their patients. Participants, who initially described negative experiences and stating that they saw no benefit, later recanted that statement by saying that if the technology were improved, they could see potential benefit. Providers, who had positive views of what telemedicine could achieve for their practice and their patients, similarly underscored that telemedicine could be improved in administration, technology and support.

### Building patient–provider relationships

Both patients and providers strongly endorsed the idea that the patient–provider relationship is vital to establishing a satisfactory and effective health care interaction, whether it was telemedicine-mediated or not. Most of these discussions focused on the broader issue of building a positive and productive patient–physician relationship foundation and issues of cultural competency, rather than specific details of how telemedicine helped or hampered relationship building. Three subthemes from these discussions included quality communication, cultural awareness/sensitivity, and demonstrated respect and caring.

Patients stressed the importance of being comfortable speaking to their primary care providers as well as the importance of providers listening to their concerns. Patients commented on the differences in language, specifically the use of medical terminology and its translation into clear messages. One participant in a patient group stated:

Like, if you say a big word to one of the elders, by the time they're thinking of what the big word is and doctor keep talking, he's not really listening. The doctor's trying to figure out, because we don't speak right away. We process before we talk.

Some members of the Alaska patient focus groups expressed concern about language differences being a barrier to effective communication between elderly patients and their primary care providers.

Providers at both sites stressed the importance of having some socio-cultural understanding of the community they were serving, including broad cultural awareness and cultural competency training to understand communication practices of patients. Providers also discussed how assumptions about cultural and communication styles can be misinterpreted and may pose as an additional barrier to providing quality care. They suggested that this could be overcome by actively seeking clarification from patients and verifying that patients share a mutual understanding of their care with their providers.

Patients additionally addressed respect and caring within the patient–provider interaction. This was a critical issue in both telemedicine-mediated encounters and face-to-face office visits. Generally, patients agreed that providers taking time to talk with patients, not rushing through the visit, and, as mentioned above, verifying understanding of what has been communicated, were ways in which genuine concern and caring were demonstrated.

Some patients specifically referenced negative experiences with providers as illustrations of “faux-pas” in clinical interactions. In both the Alaska and Hawai'i groups, participants made mention of indigenous peoples being treated in a condescending manner by health care providers who were not from their communities.

Years ago I used to encounter this all the time: doctors talked down to Natives, and, of course, a Native is not going to open up to someone that does not treat them good or talk with respect to them … it's getting better, but it needs to be getting a lot better.

This quote highlights the importance of relationships to community members which may requires individuals to overcome linguistic, cultural, socio-economic, and historical differences. Both providers and patients agreed that efforts to establish good communication and relationships were valuable to the overall health care encounter.

### Elements of an acceptable telemedicine health care model

Although no specific plans were shared about new telemedicine programs during the study, patients and providers at both sites discussed ways to improve the acceptability and effectiveness of the delivery of telemedicine. The 2 fundamental elements were that: (a) initial visits with patients must be face-to-face, and (b) patients must see the same provider on follow-up visits.

In general, providers agreed that the initial patient visit should be in-person. This belief was based on 2 perceptions: (a) establishing a relationship with a patient was accomplished faster and more effectively in-person; and (b) a thorough clinical assessment was necessary. Providers emphasised doing the initial diagnosis of current problems in-person then follow-up via telemedicine visits. One primary care provider with telemedicine experience stated:

You know it's not something where you step into a pod and you know, talk to somebody that you never talked to before. I really value that one on one [in-person] relationship, too. I think if we start from there, then we can [do telemedicine visits] and we are much more likely to be successful.

One provider also commented that seeing a patient face-to-face in their own community was a quality of care issue that enhanced their ability to provide care. Patients in Hawai'i, some familiar with telemedicine, also preferred the initial visit with a provider be face-to-face. Providers did acknowledge some initial contacts could effectively be made using telemedicine technology; however, more time should be allotted for the initial encounter when subsequent encounters are made via telemedicine.

Providers also emphasised that provider continuity in patient care is an important factor in the acceptability and potential efficacy of health care provided through telemedicine technology. Providers stated that offering continuity of care often means that patients perceived the providers as being invested in their care. Similarly, patients indicated that the continuity of seeing the same provider over time assists in open communication.

## Discussion

Notwithstanding geographic and cultural differences, AN and NH groups as indigenous populations, share similar social determinants of health and similar health disparities. In this study, both groups emphasised the access to care offered by telemedicine. Despite the use of “real time” video teleconferencing commonly used within the Hawai'i site and the “store and forward” telemedicine use at the Alaska site, participants in all focus groups recognised the benefit of using telemedicine technology to bridge the physical distances between primary care providers and rural patients. Yet the technology was not sufficient to address the “social distances” (14) between patients and providers, especially in the presence of differences in cultures, languages and ways of conceptualising concepts of health and wellness. Miller (14) commented the effects of “social distance” on patient–provider communication were greater in telemedicine due to the increased likelihood that rural patients and the consulting providers come from dissimilar backgrounds and life circumstances. Similarly, in communities of Native peoples, social distance may be magnified by cultural differences between health care providers and their patients.

The 2 elements identified as critical for creating an acceptable model of telemedicine – initial face-to-face visits and provider continuity – both refer to aspects of the patient–provider relationship, rather than features of the technology. This finding highlights the importance of patient–provider relationships in the provision of care in both indigenous populations. While previous studies have examined patient satisfaction (15) and provider acceptance/adoption of telemedicine (16), to the authors’ knowledge, only one article has explored patient–provider relationships, and its focus was on telepsychiatry (17).

It is noteworthy that both providers and patients from 2 rural Native communities in the United States endorsed the important role of the provider and patient relationship in using telemedicine. Although telemedicine was considered as having the potential to improve the quality and effectiveness of health care, the acceptability of such a model of care seemed conditional on whether and how it could nurture and enhance the patient–provider relationship. Development and maintenance of mutual, respectful, trusting relationships was noted as a foundation of a face-to-face or telemedicine-mediated clinical encounter; thus, heightened knowledge and sensitivity to the context of the people being served by health care providers is recommended.

Our study has several limitations. These findings were drawn from a small sample of patients and providers at only 2 institutions serving Native populations and have limited generalisability. In addition to the geographic and climatic differences, the 2 Native populations’ cultural values may also differ. Furthermore, the patient participants did not reflect the general population as over 3 quarters of patients reported having an education level of college or greater. Within the Hawai'i site, recruitment of patient/caregiver participants occurred through the Na Pu'uwai's diabetes education programs, with a potential over-representation of more health conscious or less well individuals. Finally, fewer than half of providers reported experience with telemedicine.

Despite these limitations, our results may inform policymakers and public health officials who are interested in improving health care access to rural and/or minority populations across the United States. Our results suggest that consideration of the human element into the telemedicine technology model holds the key to sustainable and effective use of telemedicine in remote areas for chronic disease management. Future studies are needed that will advance understanding of how to best use technology to enhance human interactions. Further understanding of the different modes of telemedicine, patient attitudes towards the use of telemedicine and differences in patient outcomes are needed. In the current milieu of digital technology and social networking, the use of telemedicine may be a promising technology, if implemented to enhance personal relationships rather than replace them.

## References

[1] Raza T, Joshi M, Schapira RM, Agha Z (2009). Pulmonary telemedicine – a model to access the subspecialist services in underserved rural areas. Int J Med Inform.

[2] Sevean P, Dampier S, Spadoni M, Strickland S, Pilatzke S (2008). Patients and families experiences with video telehealth in rural/remote communities in Northern Canada. J Clin Nurs.

[3] Hudson HE (2005). Rural telemedicine: lessons from Alaska for developing regions. Telemed J E Health.

[4] University of Alaska Anchorage Alaska Center for Rural Health (2011). Rural/Frontier, About Alaska. http://acrh-ahec.uaa.alaska.edu/frontier/alaska.html.

[5] Wiles G (2010). Hawaii 9-1-1: doctor shortage worse than recently reported.

[6] Summit focuses on Hawaii doctor shortage (2010). Pacific Business News.

[7] US Department of Health and Human Services OoMH (2011). Data/Statistics. http://minorityhealth.hhs.gov/templates/browse.aspx?lvl=1&lvlID=2.

[8] Miller EA (2003). The technical and interpersonal aspects of telemedicine: effects on doctor-patient communication. J Telemed Telecare.

[9] Kaholokula JK, Saito E, Mau MK, Latimer R, Seto TB (2008). Pacific Islanders’ perspectives on heart failure management. Patient Educ Couns.

[10] Kamaka ML (2010). Designing a cultural competency curriculum: asking the stakeholders. Hawaii Med J.

[11] Vogler J, Altmann TK, Zoucha R (2010). Native Hawaiian attitudes of culturally sensitive healthcare provider traits and behaviors. J Cult Divers.

[12] Kokesh J, Ferguson AS, Patricoski C (2004). Telehealth in Alaska: delivery of health care services from a specialist's perspective. Int J Circumpolar Health.

[13] Attride-Stirling J (2001). Thematic networks: an analytic tool for qualitative research. Qual Res.

[14] Miller EA (2002). Telemedicine and doctor-patient communication: a theoretical framework for evaluation. J Telemedicine Telecare.

[15] Mair F, Whitten P (2000). Systematic review of studies of patient satisfaction with telemedicine. BMJ.

[16] Tudiver F, Wolff LT, Morin PC, Teresi J, Palmas W, Starren J (2007). Primary care providers’ perceptions of home diabetes telemedicine care in the IDEATel project. J Rural Health.

[17] Shore JH, Savin DM, Novins D, Manson SM (2006). Cultural aspects of telepsychiatry. J Telemedicine Telecare.

